# Cross-Section Observational Study to Assess Antimicrobial Resistance Prevalence among Bovine Respiratory Disease Bacterial Isolates from Commercial US Feedlots

**DOI:** 10.3390/antibiotics12020215

**Published:** 2023-01-19

**Authors:** Erin Jobman, Jacob Hagenmaier, Nathan Meyer, Lee Bob Harper, Lisa Taylor, Kip Lukasiewicz, Dan Thomson, James Lowe, Shane Terrell

**Affiliations:** 1 Production Animal Consultation, P.O. Box 41, Scott City, KS 67748, USA; 2 Department of Veterinary Clinical Medicine, University of Illinois at Urbana-Champaign, 2001 Lincoln Ave., Urbana, IL 61802, USA; 3 Veterinary and Biomedical Research Center, 9027 Green Valley Dr., Manhattan, KS 66502, USA; 4 Boehringer Ingelheim Animal Health USA, 3239 Satellite Blvd NW, Duluth, GA 30096, USA; 5 Zoetis, 10 Sylvan Way, Parsippany, NJ 07054, USA

**Keywords:** beef cattle, susceptibility testing, epidemiology, *Pasteurella multocida*, *Mannheimia haemolytica*, *Histophilus somni*, tetracycline

## Abstract

Antimicrobial resistance (AMR) is a global public health threat that jeopardizes efficacy of antibiotics in veterinary and human medicine. Antibiotics are commonly administered to target the bacterial component of bovine respiratory disease (BRD). The objectives of this study were to obtain a better understanding of antibiotic resistance in BRD-associated bacteria (*Mannheimia haemolytica, Pasteurella multocida*, and *Histophilus somni*), investigate the clinical significance of AMR by monitoring clinical outcomes, and determine if regional differences exist in AMR trends. Deep pharyngeal swabs were used to sample beef cattle at initial BRD diagnosis (*n* = 453) from US feedlots representing three geographic regions. Organisms were identified by bacterial culture and subjected to broth microdilution antimicrobial susceptibility testing. Bacterium prevalence include *P. multocida* (36.0%), *M. haemolytica* (32.7%)*,* and *H. somni* (28.5%). Of the *Histophilus* isolates, 39.5% were resistant to at least one antimicrobial, compared to 11.7% and 8.8% *Pasteurella* and *Mannheimia*, respectively. Non-susceptibility across all organisms was 5.7 X more likely in animals that received metaphylaxis, than those that did not (*p* < 0.0001; OR 5.7; CI 2.6–12.5). During days on feed 21–40, non-susceptibility of *Histophilus* was 8.7 X more likely than *Mannheimia* (*p* = 0.0002; OR 8.7; CI 2.8 to 27.4) and 6 X more likely than *Pasteurella* (*p* = 0.0016; OR 6.0; CI 2.0–18.0).

## 1. Introduction

Bovine respiratory disease (BRD) is the leading cause of feedlot morbidity and mortality, arising from complex interactions between pathogens, environmental stress, and host factors. BRD accounts for 70–80% of total morbidity and 10–50% mortality in US feedlots [1]. The cattle industry suffers consequences of BRD due to medication costs, mortality, and subsequent loss in health performance. Illness often occurs post-arrival into the feedlot following various stressors to the animal’s immune system such as weaning, comingling, dietary transitions, and transportation [1,2].

Multiple viral agents can contribute to the development of BRD including bovine viral diarrhea virus (BVDV), bovine respiratory syncytial virus (BRSV), parainfluenza-3 virus (PI3), and bovine herpes virus-1 (BHV-1). The combination of viruses, host stress, and environmental changes can lead to enhanced colonization and infection of bacterial species such as *Mannheimia haemolytica*, *Pasteurella multocida*, *Histophilus somni,* and *Mycoplasma bovis* [1,2,3,4,5]. These organisms are commonly targeted in commercial vaccines; however, the main bacteria genera are observed in both healthy and BRD-affected animals [2,3,6]. Opportunistic characteristics of these bacteria complicate a clear understanding of pathogenesis [2,7,8,9].

Due to the bacterial component of BRD, antibiotics are valuable tools for treatment and control. In North America, antimicrobials are commonly administered at or soon after arrival at the feedlot for BRD control (i.e., metaphylaxis), and/or at the time of illness diagnosis for treatment [5,10]. Feed-grade antibiotics can also be used for treatment of respiratory disease. Maintaining the efficacy of these drugs is a highly complex issue, affecting both human and animal health, thus, it is a significant priority for livestock producers, veterinarians, and consumers.

Pragmatic strategies to monitor antimicrobial resistance (AMR) among BRD pathogens are generally lacking in the United States. Surveillance for cattle exists only for enteric pathogens and is reported by the National Antimicrobial Resistance Monitoring System (NARMS), a collaborative project among the Centers for Disease Control and Prevention (CDC), United States Department of Agriculture (USDA), and Food and Drug Administration (FDA) [11]. Veterinary diagnostic laboratories have reported general trends of decreasing antimicrobial susceptibility [5,10,12]; however, it is unclear how the data compares to the general cattle population due to potential sampling bias. The data is often generated from lung tissue of fatal BRD cases, likely animals treated with antibiotics at least once [10,12].

To support veterinarians in their efforts towards optimizing antibiotic efficacy, it is critical to understand the resistance prevalence among bacterial pathogens at the time of initial BRD diagnosis.

The objectives of this study were to (i) quantify phenotypic antibiotic resistance prevalence of BRD-associated bacteria prior to treatment following initial diagnosis in feedlots, (ii) understand the clinical significance of AMR obtained through culture and sensitivity by monitoring treatment outcomes, and (iii) determine if clinically relevant differences in AMR exist by geographic regions of calf origin or feedlot location.

## 2. Results

### 2.1. Cattle Health and Performance

From the total animals sampled (*n* = 469), 453 animals remained in the study due to eligibility criteria. Of those, 181 were heifers and 272 were steers. Seventy-eight calves (17.2%) received parenteral metaphylaxis. Feed-grade antibiotics were administered to 336 (74.2%) animals, all of which received chlortetracycline. Animals that received both metaphylaxis and feed-grade antibiotics totaled 69 head (15.2%). All samples were collected at the first diagnostic case identification of BRD for that individual animal. Days on feed (DOF) was recorded at this time and ranged from 1 to 253, median of 35 (Figure S1). Weights were also recorded and ranged from 306 to 1574 pounds, median of 809 pounds (Figure S2). All animals in the study received antimicrobial treatment following sample collection. Antibiotics were administered according to the product’s label directions at the discretion of the feedlot. Overall mortality totaled 32 calves (7.1%), three of which died due to causes other than respiratory disease, resulting in a BRD case fatality rate of 6.4%. In total, 110 animals required additional treatment for BRD, 15 of which died from BRD and 13 deviated from the lot shipment date. Treatment success was defined as a calf that does not require additional treatment, nor dies, nor deviates from the lot shipment date. Animals that were not retreated totaled 343, 14 of which died from BRD, and 9 deviated from the lot shipment date, leading to a first treatment success rate of 70.2%. Total outcomes included 399 animals (88.0%) shipped to slaughter with their respective lot while 22 animals (4.9%) deviated from the lot shipment date in the form of early (11), late (2), or railed (9) shipments.

### 2.2. Bacterial Isolation

Bacterial culture generated 20 “No growth” and 114 “No significant growth” results. No significant growth means that bacterial organisms other than the species of interest were detected. Together, these were combined in the analysis as “Negative” and totaled 134 observations from 453 animals (29.6%). Bacteriology results include 129 *Histophilus somni* isolates (28.5%), 148 *Mannheimia haemolytica* (32.7%) and 163 *Pasteurella multocida* (36.0%).

Two hundred and fourteen animals (67.1%) yielded one organism, 89 (27.9%) yielded two organisms, and 16 (5.0%) yielded all three organisms. Of the animals with two isolates, most commonly *P. multocida* + *M. haemolytica* were isolated together (45 animals, 14.1%), then *P. multocida* + *H. somni* (29 animals, 9.1%), and *H. somni* + *M. haemolytica* (15 animals, 4.8%).

A chi-squared analysis was used to detect associations between bacterial organism and categorical metadata collected. A significant association was detected between metaphylaxis usage and organism prevalence (χ^2^ = 10.44; *p* = 0.0054). A significant relationship was also detected between metaphylaxis and animals requiring retreatment for BRD (χ^2^ = 12.99; *p* = 0.0003).

### 2.3. Antimicrobial Susceptibility

**Figure 1:**
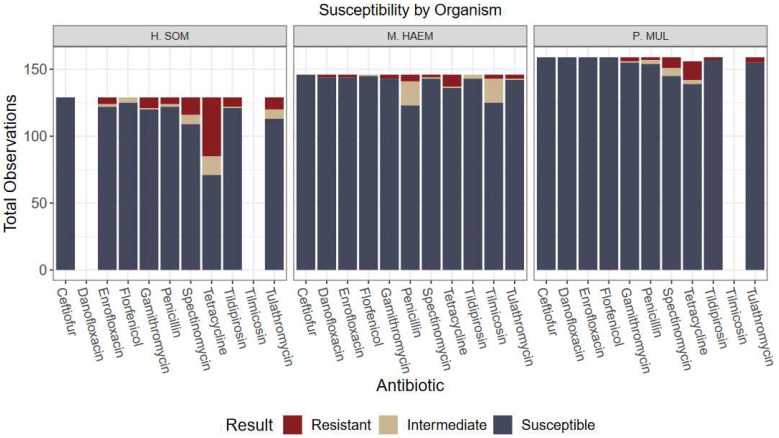
Culture results grouped by organism and antibiotic susceptibility. Only antibiotics with CLSI-approved breakpoints for BRD were included in analysis. Greatest resistance observed was *H. somni* to tetracycline (34.1% resistance).

Of the total *H. somni* isolates, 51 of 129 (39.5%) were resistant to at least one antimicrobial. Most notably, 44/129 (34.1%) of *H. somni* isolates were resistant to tetracyclines (Figure 1, Table S1). Additional resistance was greatest to spectinomycin (10.1%), tulathromycin (7.0%), and gamithromycin (6.2%). Multidrug resistance (MDR) is defined as an isolate that is not susceptible to at least one agent in 3 or more antimicrobial classes [13]. According to this definition, 12/129 (9.3%) of *Histophilus* isolates were multidrug resistant. Isolates susceptible to all antibiotics totaled 65/129 (50.4%).

For *M. haemolytica*, 13 of 148 (8.8%) isolates were resistant to at least one antimicrobial and 4/148 (2.7%) isolates were multidrug resistant. These isolates were most commonly resistant to tetracycline (6.1%) and penicillin (3.4%). For *P. multocida*, 19 of 163 (11.7%) were resistant to at least one antimicrobial and 3/163 (1.8%) were MDR. *P. multocida* isolates were mostly resistant to tetracycline (8.6%) and spectinomycin (4.9%). Isolates susceptible to all antibiotics totaled 103/148 (69.6%) for *M. haemolytica* and 132/163 (81.0%) for *P. multocida* (Figure 1, Table S1).

Resistance to at least one macrolide was 7.0% for *H. somni*, 2.0% for *M. haemolytica*, and 2.5% for *P. multocida*. Of these observations, 50% had received metaphylaxis, 75% of which used a macrolide for metaphylaxis.

### 2.4. Effects of Metadata on Prevalence and Resistance of Bacterial Organisms Using Generalized Linear Mixed Models

**Figure 2:**
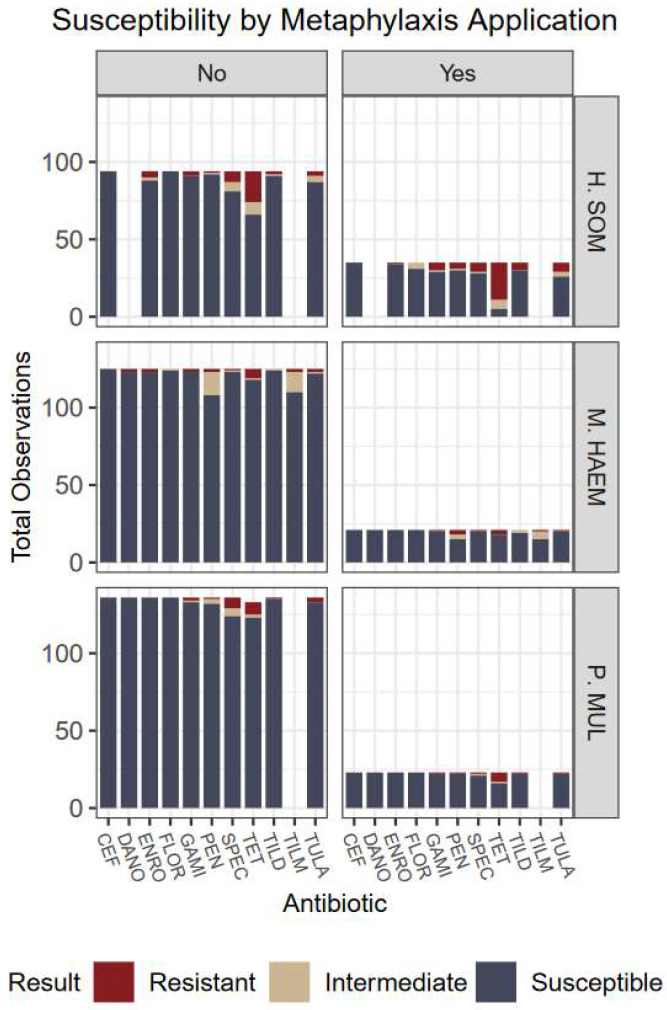
Culture results grouped by organism, susceptibility, and metaphylaxis usage. No indicates the animal did not receive metaphylaxis; animals that received metaphylaxis are represented by Yes. Antibiotics: CEF: ceftiofur; DANO: danofloxacin; ENRO: enrofloxacin; FLOR: florfenicol; GAMI: gamithromycin; PEN: penicillin; SPEC: spectinomycin; TET: tetracycline; TILD: tildipirosin; TILM: tilmicosin; TULA: tulathromycin.

**Figure 3:**
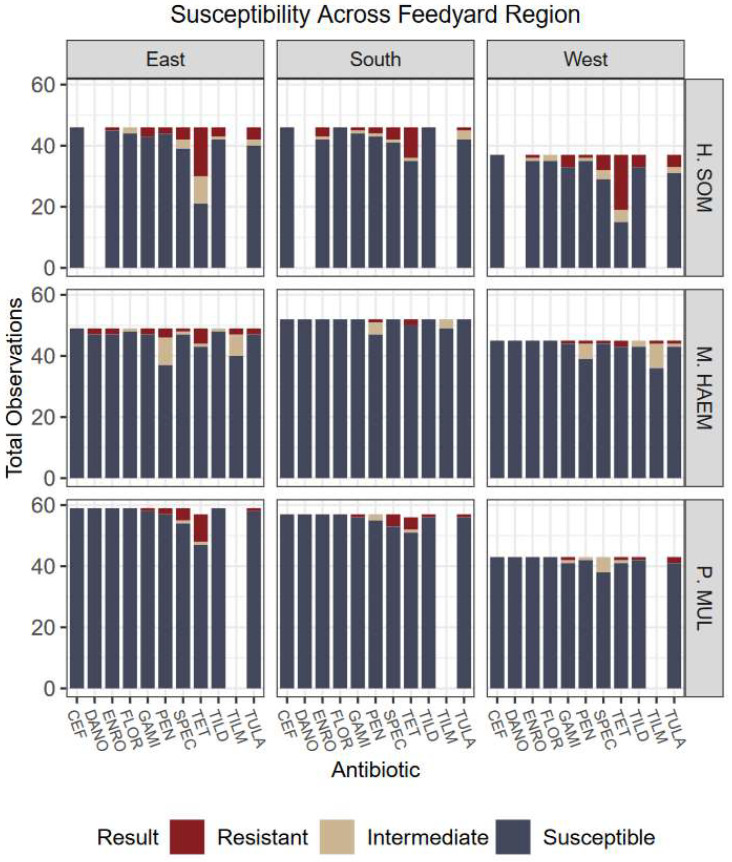
Culture results grouped by organism, susceptibility, and geographic feedlot region. Five feedlots represent each region. Regions were defined by the authors and represent areas of Colorado, Kansas, and Nebraska. Evidence did not support associations of region influencing cattle outcomes nor bacteria prevalence. Antibiotics: CEF: ceftiofur; DANO: danofloxacin; ENRO: enrofloxacin; FLOR: florfenicol; GAMI: gamithromycin; PEN: penicillin; SPEC: spectinomycin; TET: tetracycline; TILD: tildipirosin; TILM: tilmicosin; TULA: tulathromycin.

**Figure 4:**
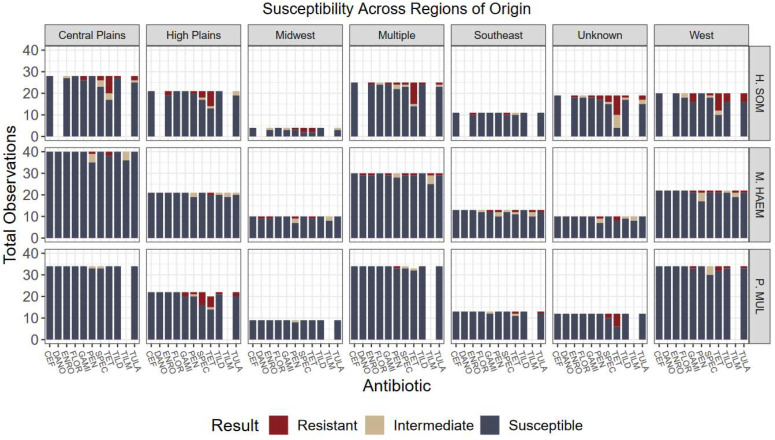
Culture results grouped by organism, susceptibility, and geographic region of origin. Five regions were designated by the authors to represent the states where calves originated from. No statistical differences were detected among variables and region of origin. Antibiotics: CEF: ceftiofur; DANO: danofloxacin; ENRO: enrofloxacin; FLOR: florfenicol; GAMI: gamithromycin; PEN: penicillin; SPEC: spectinomycin; TET: tetracycline; TILD: tildipirosin; TILM: tilmicosin; TULA: tulathromycin.

Overall, the lack of variation among antimicrobial susceptibility results can be visualized in Figure 1, Figure 2, Figure 3 and Figure 4. Differences in the proportion of susceptible and non-susceptible (intermediate + resistant) observations were evaluated in multiple analyses. Susceptibility proportions by bacterial species generally followed overall outcome trends described previously (88% shipped, 4.9% deviated, 7.1% mortality, and 70.2% first treatment success) (Table 1). Greatest resistance was observed to tetracyclines and was further explored with generalized linear mixed models.

Examining non-susceptibility of tetracyclines, the overall type III F-test for the interaction between organism and metaphylaxis was not significant (*p* = 0.14). However, individual main effects were significant (*p* < 0.0001 each). Non-susceptibility to tetracyclines was 18 X more likely in *Histophilus* than *Mannheimia* (*p* < 0.0001; OR = 18.3; CI 7.06–47.2) and 9 X more likely than *Pasteurella* (*p* < 0.0001; OR = 9.2; CI 4.1–20.4). Proportion of non-susceptibility was also significantly different (*p* < 0.0001) amidst usage of metaphylaxis groups. Non-susceptibility to tetracyclines across all organisms was 5.7 X more likely in animals that received metaphylaxis, compared to those that did not (*p* < 0.0001; OR 5.7; CI 2.6–12.5). Susceptibility results with and without implementation of metaphylaxis can be visualized in Figure 2.

In a different model analyzing tetracycline non-susceptibility, the overall type III F-test for the interaction between organism and days on feed groups (*n* = 5 groups; ~20-day increments) was significant (*p* < 0.0001). Group 2 (DOF 21–40), non-susceptibility of *Histophilus* was 8.7 X more likely than *Mannheimia* (*p* = 0.0002; OR 8.7; CI 2.8 to 27.4) and 6 X more likely than *Pasteurella* (*p* = 0.0016; OR 6.0; CI 2.0–18.0). In group 3 (DOF 41–60), non-susceptibility of *Histophilus* was 6.4 X more likely than *Pasteurella* (*p* = 0.0016; OR 6.4; CI 2 to 20.4).

In the model evaluating non-susceptibility of tetracyclines to metaphylaxis and retreated animals, an overall type III F-test of fixed effects was significant (*p* = 0.0467). However, the simple effects of this model demonstrate no significant difference in proportion of resistance of animals requiring retreatment among animals that received metaphylaxis (*p* = 0.1263).

Overall, no significant associations were detected in the proportion of non-susceptibility among feedlot regions (*p* = 0.2226) nor calf origins (*p* = 0.0668); however, an association may exist had less data been designated to “unknown” and “multiple” categories or larger sample size used (Figure 3 and Figure 4). Further, associations were not detected among relationships of usage of feed-grade antibiotics and outcomes (*p* = 0.6437).

## 3. Discussion

This study describes the prevalence of bacterial species and culture sensitivity results from cattle in commercial US feedlots sampled at initial diagnosis of BRD. The most frequent bacterium isolated from deep pharyngeal swabs was *P. multocida*, followed by *M. haemolytica*, and *H. somni*. These results are consistent with lower respiratory tract findings as demonstrated by Timsit et al. [3] and upper respiratory tract findings [14,15]. Resistance to at least one antimicrobial was lower for *M. haemolytica* (8.8%) and *P. multocida* (11.7%), compared to *H. somni* (39.5%). A case–control study by Timsit et al. reports higher prevalence of resistance to at least one antimicrobial for all three bacteria isolated from morbid and healthy controls: *M. haemolytica* 84.6%, *P. multocida* 92.6%, and *H. somni* 66.7% [3]. In both studies, samples for culture and sensitivity were collected at the time of first BRD diagnosis; however, Timsit’s study collected trans-tracheal aspirations from the lower respiratory tract. While cattle in these studies may not be compared equally, the differences suggest spatial dynamics in resistance profiles among the upper and lower respiratory tract in cattle affected with BRD at similar time points. The island model has been used in human medicine to study this concept of respiratory microbiome dispersion [2]. A different study investigating resistance to *Mannheimia* also reports higher resistance to at least one antimicrobial (37%) from deep pharyngeal swabs (DPS) collected at initial BRD diagnosis than what is demonstrated in this study [8]. Overall, resistance to tetracycline and members of the macrolide class identified by susceptibility results was the most common and consistent finding in the literature [3,8,10,11,12,13,14,15].

A three-year retrospective analysis conducted by Kansas State University Veterinary Diagnostic Lab detected up to 82.7% of Mannheimia isolates were resistant to at least one antimicrobial [12]. This study included lung tissue isolates from deceased animals. Using resistance to three or more antimicrobials as their definition for multidrug resistance, 42%, 46%, and 63% of the isolates were classified as multidrug resistant in each year analyzed. Sampling at initial BRD diagnosis generated much lower MDR isolates (*M. haemolytica* 2.7%; *P. multocida* 1.8%; *H. somni* 9.3%) utilizing the MDR definition described previously.

A different report described cases submitted to Iowa State University Veterinary Diagnostic Lab and analyzed with prior treatment history and antimicrobial resistance from lower respiratory tract samples. They reported across all three bacteria species (*M. haemolytica*, *P. multocida*, and *H. somni*) the percentage of resistant isolates increased as the number of antimicrobial treatments increased (χ^2^ = 93; *p* < 0.0001) [16]. Previously untreated animals demonstrated MDR in less than 5% of total isolates compared to 47% from animals with three or more previous antimicrobial treatments [16].

Although the results from these diagnostic laboratories do not represent animals with BRD that survived, judicious antimicrobial usage must be a priority to maintain efficacy. Diagnostic laboratory trends and susceptibility profiles presented here should be considered together to appreciate chronological changes occurring as sick animals fail to recover.

This dataset illustrates differences in resistance among animals that received metaphylaxis. Greater resistance for *Mannheimia* was also demonstrated following metaphylaxis administration in calves [17]. Potential risks of commercial practices to unintentionally select for resistance should be considered. Resistant *Histophilus* isolates were more likely to be identified from animals sampled between 20 to 40 days on feed. This finding provides insight on timing of *Histophilus* isolation associated with BRD morbidity; however, more research is needed to accurately comment on infection dynamics across time.

The prevalence of one bacterial species over another did not affect BRD outcomes (Table 1). Given the lack of resistance overall, resistance phenotypes were not associated with differences in outcomes. A cross-sectional cohort study with 1026 heifers also reported poor association between BRD clinical outcomes and susceptibility to tulathromycin [18]. There was no evidence to support any associations of bacterial prevalence nor resistance phenotypes among geographic regions, when considering regions of the feedlots and of calf origins. Associations may exist in a larger sample size or dataset with more variation among resistance and outcomes.

There are several limitations to this study. Large odds ratios and wide confidence intervals generated by mixed model regression limit the precision to which conclusions can be drawn from this study. Current methods for diagnosis and prognosis of BRD still have low accuracy [2]—30% of culture results did not yield a result when taken from clinically abnormal animals. This could be attributed to viral causes of BRD and/or lack of sensitivity of diagnostics. Similar findings exist in the literature from cattle with BRD [3,8,18]. After decades of BRD research, presence of bacteria in the respiratory tract remains elusive towards outlining a clear causal link to pathogenesis. *Mannheimia*, for example, has 12 capsular serotypes with varying degrees of pathogenicity [7,8]. Lastly, antimicrobial susceptibility testing does not guarantee a specific clinical result in an individual animal; therefore, it is difficult to draw conclusions of animal outcomes based on susceptibility results [5,18]. Ultimately, disease outcome is influenced by factors that fluctuate among individual animals such as host immune status, genotype of host and pathogens, stress factors, disease severity and duration, etc., which were not accounted for in this study.

## 4. Materials and Methods

### 4.1. Ethics Statement

All cattle handling and treatment procedures involved in conducting this study followed the Guide for the Care and Use of Agricultural Animals in Research and Teaching [19].

### 4.2. Animals

Geographic locations of US feedlots and cattle origin were of interest to determine if AMR trends exist regionally. To account for this, feedlot and cattle origin locations were collected separately. Cattle enrollment locations were clustered into 3 geographic regions within Colorado, Kansas, and Nebraska as defined by the authors. Fifteen feedlots were enrolled; five feedlots are represented per region. Origins were defined separately based on where the cattle were shipped from prior to entering the feed yard. Five regions of origin were designated by the authors and are represented by the following: West: Washington, Oregon, Montana, Idaho, Wyoming, Colorado, Utah, Nevada, California, Arizona, and New Mexico; High Plains: Texas and Oklahoma; Central Plains: Kansas, Nebraska, South Dakota, and North Dakota; Midwest: Minnesota, Iowa, Missouri, Illinois, Wisconsin, Indiana, Michigan, and Ohio; Southeast: Arkansas, Louisiana, Mississippi, Alabama, Georgia, Florida, South Carolina, North Carolina, Virginia, West Virginia, Tennessee, and Kentucky.

Trained personnel employed by the feedlots evaluate cattle health daily. Potential respiratory disease cases are visually identified and brought to the hospital for closer examination and treatment. The cattle enrolled in this study are a subset of each feed yard’s typical daily pull population. A minimum of ten samples were collected during each sampling day and each feed yard was collected a minimum of 3 times to achieve the desired sample size. Using a described equation for calculating sample size for prevalence studies with a targeted precision of 15%, a minimum of 160 isolates were required each of *Mannheimia haemolytica*, *Pasteurella multocida*, and *Histophilus somni* [20,21]. Assuming routine culture recovery to be 35% per species, a total of 450 cattle were required to be enrolled in the study. To ensure each feedlot region (*n* = 3) was equally represented, a minimum of 150 cattle were sampled per region.

### 4.3. Eligibility

Cattle originating from Mexico and/or dairy-influenced cattle were not eligible for enrollment. BRD case definition was defined as animals generating a Whisper^®^ Lung Score > 1 (scale 1–5), rectal temperature ≥ 104° F, and presence of one or more clinical signs: increased respiratory rate, dyspnea, lethargy, depression, inappetence, dehydration, and ocular or nasal discharge. Cattle determined to be affected by BRD by feedlot caregivers were further examined by a veterinarian (JH) for confirmation, screening, and sampling. Cattle with any pre-existing abnormal health conditions were not eligible for enrollment. Further, any cattle with comorbidities such as infectious pododermatitis or primary metabolic disturbances were not eligible for sampling.

Non-controlled feed yard data suggest similar efficacy when the same antibiotic is used for metaphylaxis and first treatment, compared to alternative antibiotics. This has been observed when metaphylaxis and first treatment occur at least 21 days apart (J. Hagenmaier and S. Terrell, personal communication, October 2020). Therefore, cattle that received metaphylaxis were eligible for enrollment if 21 days had passed since administration. However, cattle sampled on a given day with a history of metaphylaxis were not permitted to exceed 20% to avoid unintended overrepresentation. Pens that received a feed-grade antibiotic at any point during the feeding period were eligible for enrollment.

### 4.4. Sample Collection

Deep pharyngeal swabs (DPS) were obtained from eligible cattle prior to receiving antibiotic treatment. At the time of sampling, additional data captured include gender, days on feed, rectal temperature, lung score, body weight, treatment drug, prior metaphylaxis use and feed-grade antibiotics received, if applicable.

Deep pharyngeal swabs were collected with a 33-inch double guarded, sterile uterine swab from cattle at time of diagnosis prior to treatment. To collect, the calf was properly restrained in the chute and the nares cleaned with a disposable wipe. The swab was carefully inserted into the ventral meatus and advanced until approximately halfway between the external nares and medial canthus of the eye. At this point, the swab was advanced through the protective guard and rotated against the pharyngeal wall for a minimum of 5 rotations. The swab was then retracted into the plastic guard and removed from the animal safely. Swabs were submerged into tubes containing liquid AMIES medium and placed on ice for overnight shipment to the Iowa State Veterinary Diagnostic Laboratory.

Clinical outcomes were recorded for each calf enrolled in the study until harvest. Feed yard electronic health management software was used to record subsequent treatment, if any, in addition to mortality or deviations from lot shipment date. Treatment success was defined as a calf that does not require additional treatment, nor dies, nor is removed from the population prior to the lot shipment date.

### 4.5. Sample Processing

Samples were sent to Iowa State Veterinary Diagnostic Laboratory for culture and susceptibility diagnostics. Swabs were removed from the AMIES media and used to inoculate blood agar, 10% bovine blood agar, and Tergitol T-7 agar plates. Plates were incubated aerobically, anaerobically, and with 5% CO_2_ at 35°C for 24–48 h. Colonies with morphology consistent with *Mannheimia heamolytica*, *Pasteurella multocida*, and *Histophilus somni* were identified. Correct identification for one isolate per pathogen per sample was confirmed using matrix-assisted laser desorption ionization time-of-flight mass spectrometry (MALDI TOF) techniques following manufacturer’s specifications. If multiple colonies were isolated for a single pathogen from one sample, the lab randomly selected the pathogen to be confirmed by MALDI TOF using their standard operating procedures. Following identification using MALDI-TOF, two agar plates were prepared with a pure culture of each pathogen: one sent for antimicrobial susceptibility and the other frozen and archived.

Each confirmed isolate was subjected to broth microdilution antimicrobial susceptibility testing using the Sensititre BOPO7F Bovine/Porcine Panel (Thermo Fisher Scientific, Waltham, MA, USA). Materials involved were required to pass quality control protocols set forth by the diagnostic lab. A saline suspension of isolated colonies was adjusted to achieve turbidity equivalent to a 0.5 McFarland standard. The adjusted suspension was then diluted in broth to inoculate the BOPO7F plate. Inoculated plates incubated for 18–24 h at 37 °C and were interpreted with the manufacturer’s manual system (Sensititre Vizion system, Thermo Fisher Scientific).

Standardized methods for antimicrobial susceptibility testing and interpretation are developed by the Clinical and Laboratory Standards Institute (CLSI) [22]. The most current CLSI breakpoints reported in the 5th edition of Performance Standards for Antimicrobial Disk and Dilution Susceptibility Tests for Bacteria Isolated from Animals were utilized. CLSI-approved veterinary breakpoints apply to a specific combination of disease, pathogen, animal species, and antimicrobial treatment regimen [22]. At the time of experimentation, CLSI-approved breakpoints of BRD-associated bacteria (*Mannheimia haemolytica*, *Pasteurella multocida*, and *Histophilus somni*) included the following antimicrobials: Ampicillin, Ceftiofur, Danofloxacin (except for *Histophilus*), Enrofloxacin, Florfenicol, Gamithromycin, Penicillin G, Spectinomycin, Tetracycline, Tildipirosin, Tilmicosin (*Mannheimia* only), and Tulathromycin [22]. Results were generated as minimum inhibitory concentration (MIC) values and categorized as susceptible (S), intermediate (I), or resistant (R). CLSI defines susceptible, intermediate, and resistant as referenced in [5].

### 4.6. Statistical Analysis

The statistical analysis includes susceptibility data generated using CLSI-approved breakpoint combinations only. Ampicillin was excluded due to excess in “No interpretation” results. Data visualization was performed using ggplot2 in RStudio (R version 3.3). A chi-squared analysis was used to detect associations between bacterial organism and categorical metadata collected. Effects of metadata on prevalence and resistance of bacterial organisms was analyzed using generalized linear mixed models (PROC GLIMMIX; SAS Version 9.4). Susceptibility results were grouped into two categories: susceptible and non-susceptible (resistant + intermediate results). Differences in proportion of non-susceptible were evaluated in multiple analyses with the following categorical variables: calf origin (*n* = 7 groups), feedlot region (*n* = 3), metaphylaxis (y/n), feed-grade antibiotics (y/n), days on feed groups (*n* = 5), retreated animals (y/n), and deviations in shipping outcome (*n* = 3). All models utilized a binomial distribution to estimate the response proportions with logit log link. The model included a random effect of day nested within feed yard. Individual animal served as the experimental unit. Statistical significance was declared when *p* ≤ 0.05.

## 5. Conclusions

Antimicrobial resistance is undoubtedly a complex, global threat with public health implications. Understanding how to preserve the efficacy of antibiotics in veterinary medicine is a substantial priority. This dataset demonstrates greatest resistance to tetracyclines among all three bacteria species. Overall resistance prevalence at initial diagnosis of BRD was not high enough to correlate with health and performance outcomes; however, a larger, randomized, or longitudinal study may generate different associations. This study adds to existing information describing susceptibility trends in BRD among North American feedlots. To support veterinarians in their efforts towards optimizing antibiotic efficacy, it is critical to understand how resistance changes over time. Resistance prevalence in the literature is difficult to compare due to different sample substrates, reporting of MICs vs. categorical susceptibility, and changes in CLSI-approved breakpoints over time. Further, consistent research is needed to understand how resistance phenotypes develop to ensure antimicrobial efficacy.

## Figures and Tables

**Table 1:** Number of animals with positive culture and susceptibility results grouped by outcome categories. Susceptible reflects the number of animals that were susceptible across all antibiotics per respective culture result. Non-susceptible (Non-Susc.) reflects the number of animals non-susceptible (intermediate and/or resistant) to at least one antimicrobial per respective culture result. Org: Organism; HS: *H. somni*; MH: *M. haemolytica*; PM: *P. multocida*; Dev.: Deviated from lot shipment date; 1st TX Succ.: first treatment success; ReTX: retreated animals.

Outcomes							
Org.	Proportion	Total	Dead	Dev.	Shipped	1st TX Succ.	ReTX
HS	Susceptible	65	4 (6.2%)	2 (3.1%)	59 (90.8%)	47 (72.3%)	16 (24.6%)
	Non-Susc.	64	4 (6.3%)	5 (7.8%)	55 (85.9%)	42 (60.1%)	22 (34.7%)
MH	Susceptible	103	8 (7.8%)	6 (5.8%)	89 (86.4%)	74 (71.8%)	25 (24.3%)
	Non-Susc.	43	4 (9.3%)	0 (0%)	39 (90.7%)	33 (76.7%)	7 (16.3%)
PM	Susceptible	132	5 (3.8%)	10 (7.6%)	117 (88.6%)	97 (73.5%)	27 (20.5%)
	Non-Susc.	26	1 (3.9%)	1 (3.9%)	24 (92.3%)	21 (80.8%)	5 (19.2%)

## Data Availability

Data available upon request to corresponding author.

## Supplementary Materials

The following supporting information can be downloaded at: https://www.mdpi.com/article/10.3390/antibiotics12020215/s1, Figure S1: Days on Feed Distribution at Initial BRD Diagnosis; Figure S2: Weight Distribution at Initial BRD Diagnosis. Table S1: Culture Results by Organism and Antibiotic Susceptibility.
