# Supplementary material for: Cross-Section Observational Study to Assess Antimicrobial Resistance Prevalence among Bovine Respiratory Disease Bacterial Isolates from Commercial US Feedlots
Source: Antibiotics (Basel). 2023 Jan 19;12(2):215. doi: 10.3390/antibiotics12020215 (PMC9952279; doi:10.3390/antibiotics12020215)
Supplement: Supplementary file 1 [file antibiotics-12-00215-s001.zip › antibiotics-2147696-supplementary.pdf]

antibiotics

*Article*

# Cross-Section Observational Study to Assess Antimicrobial Resistance Prevalence among Bovine Respiratory Disease Bacterial Isolates from Commercial US Feedlots

**Erin Jobman [1,2], Jacob Hagenmaier [3], Nathan Meyer [4], Lee Bob Harper [5], Lisa Taylor [1], Kip Lukasiewicz [1], Dan Thomson [1], James Lowe [1,2] and Shane Terrell [1,*]**

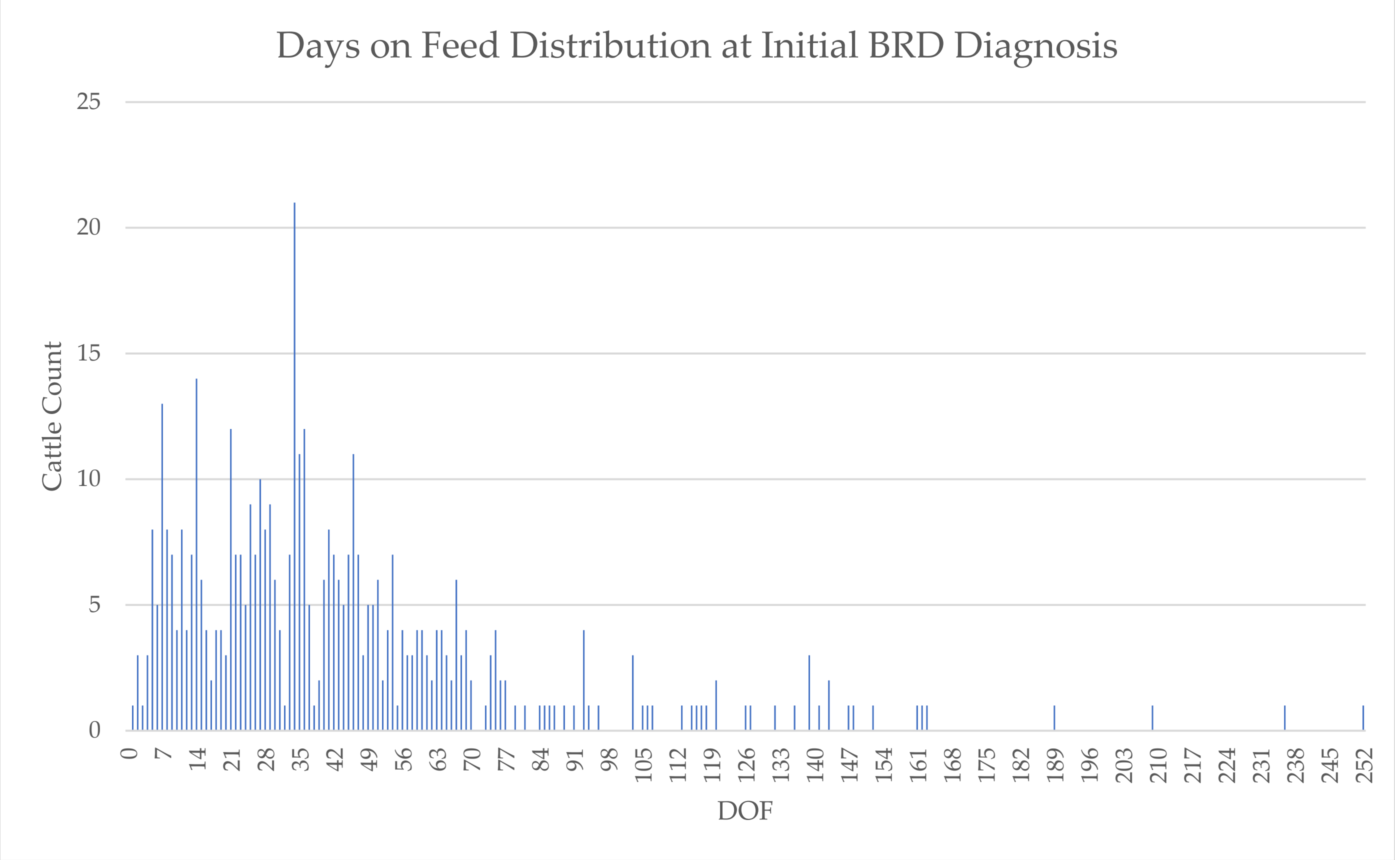


Supplementary Figure S1: Range of days on feed (DOF) of the sampled animals at initial diagnosis of BRD.

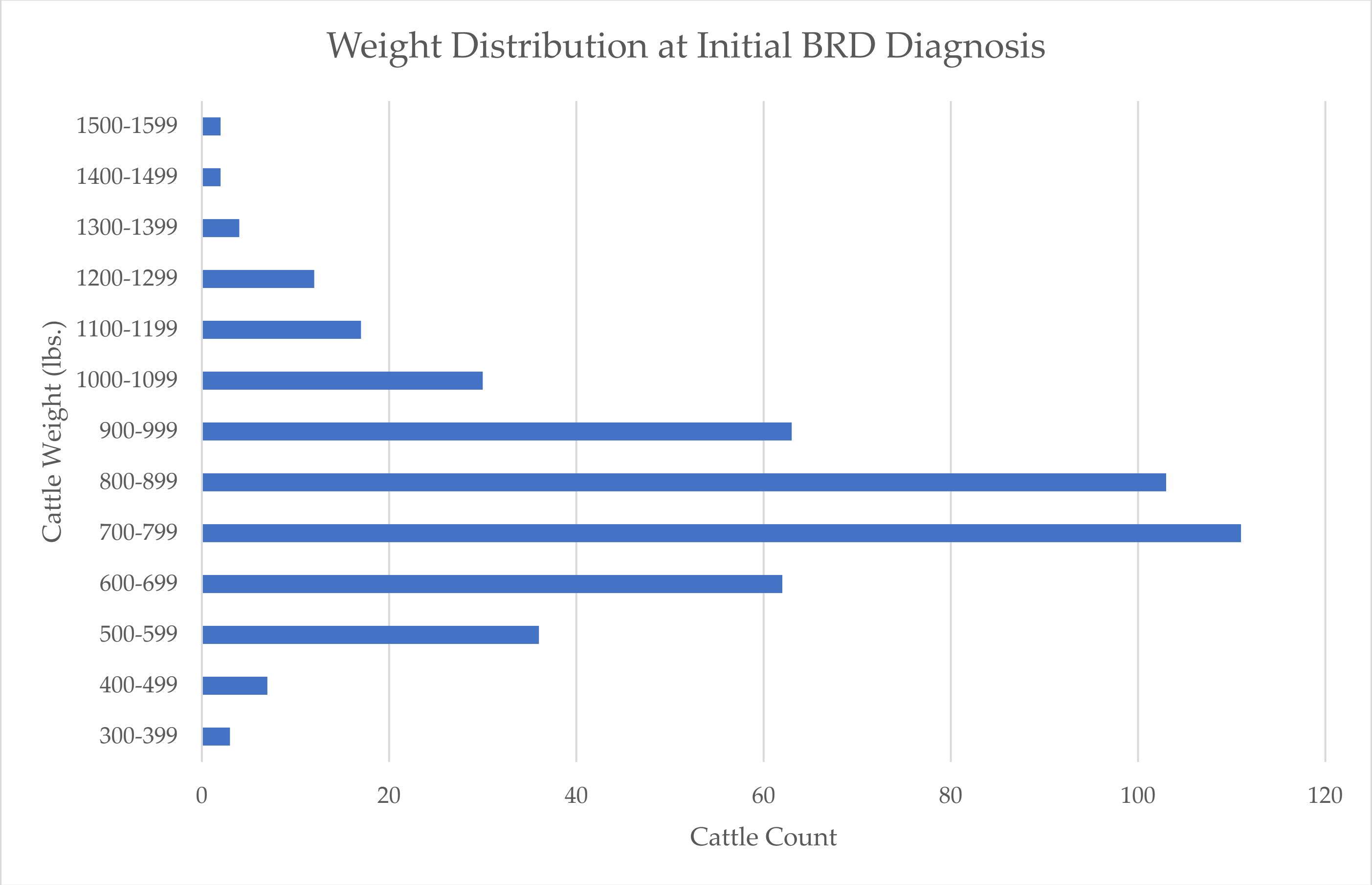


Supplementary Figure S2: Weight distribution and animal frequency at initial diagnosis with BRD.

| | H SOM | | | | M HAEM | | | | P MUL | | | |
|---|---|---|---|---|---|---|---|---|---|---|---|---|
| Antibiotic | S | I | R | R% | S | I | R | R% | S | I | R | R% |
| Ceftiofur | 129 | 0 | 0 | 0 | 146 | 0 | 0 | 0 | 159 | 0 | 0 | 0 |
| Danofloxacin | - | - | - | - | 144 | 0 | 2 | 1.4 | 159 | 0 | 0 | 0.0 |
| Enrofloxacin | 122 | 2 | 5 | 3.9 | 144 | 0 | 2 | 1.4 | 159 | 0 | 0 | 0.0 |
| Florfenicol | 125 | 4 | 0 | 0.0 | 145 | 1 | | 0.0 | 159 | 0 | 0 | 0.0 |
| Gamithromycin | 120 | 1 | 8 | 6.2 | 143 | 0 | 3 | 2.0 | 155 | 1 | 3 | 1.8 |
| Penicillin | 122 | 2 | 5 | 3.9 | 123 | 18 | 5 | 3.4 | 154 | 3 | 2 | 1.2 |
| Spectinomycin | 109 | 7 | 13 | 10.1 | 143 | 1 | 2 | 1.4 | 145 | 6 | 8 | 4.9 |
| Tetracycline | 71 | 14 | 44 | 34.1 | 136 | 1 | 9 | 6.1 | 142 | 3 | 14 | 8.6 |
| Tildipirosin | 121 | 1 | 7 | 5.4 | 143 | 3 | 0 | 0.0 | 157 | 0 | 2 | 1.2 |
| Tilmicosin | - | - | - | - | 125 | 18 | 3 | 2.0 | - | - | - | - |
| Tulathromycin | 113 | 7 | 9 | 7.0 | 142 | 1 | 3 | 2.0 | 155 | 0 | 4 | 2.5 |

Supplementary Table S1: Number of susceptible (S), intermediate (I) and resistant (R) observations from each bacterial species. Prevalence of resistance (R%) is represented for each antibiotic. H SOM: *H. somni*; M HAEM: *M. haemolytica*; P MUL: *P. multocida*.
